# Complete Genome Sequence of Pseudoalteromonas sp. Strain OCN003, Isolated from Kāne’ohe Bay, O’ahu, Hawaii

**DOI:** 10.1128/genomeA.01396-14

**Published:** 2015-01-15

**Authors:** Silvia Beurmann, Patrick Videau, Blake Ushijima, Ashley M. Smith, Greta S. Aeby, Sean M. Callahan, Mahdi Belcaid

**Affiliations:** a Department of Microbiology, University of Hawaii, Honolulu, Hawaii, USA; b Hawai’i Institute of Marine Biology, Kāne’ohe, Hawaii, USA

## Abstract

Pseudoalteromonas sp. strain OCN003 is a marine gammaproteobacterium that was isolated from a diseased colony of the common Hawaiian reef coral, Montipora capitata, found on a reef surrounding Moku o Lo’e in Kāne’ohe Bay, Hawaii. Here, we report the complete genome of Pseudoalteromonas sp. strain OCN003.

## GENOME ANNOUNCEMENT

Pseudoalteromonas is a genus of Gram-negative marine bacteria involved in mutualistic and pathogenic relationships with other marine organisms. Strains of Pseudoalteromonas colonize and influence the metamorphosis of polychaete (1), coral (2), sea urchin (3), and bryozoan (4) larvae. The production of a biofilm and the release of signaling molecules are thought to be the mechanisms that trigger settlement or metamorphosis in many of these cases. Strains of Pseudoalteromonas have also been implicated as etiological agents in several diseases of marine organisms, specifically fish (5), crustaceans (6, 7), and sponges (8).

Acute Montipora white syndrome (aMWS) is a tissue loss disease affecting a major reef-building coral, Montipora capitata, in Kāne’ohe Bay, Hawaii (9). This disease is characterized by rapid tissue loss, which can lead to total colony mortality. Here, we present the full-genome sequence of Pseudoalteromonas sp. strain OCN003, which was isolated from a diseased colony of M. capitata on a reef surrounding the island Moku o Lo’e in Kāne’ohe Bay, Hawaii. The diseased fragment of M. capitata was crushed and plated on glycerol artificial seawater (GASW) agar (10). Genomic DNA from an axenic culture of OCN003 was isolated using a phenol-chloroform extraction method and sequenced using the PacBio RS II system at the University of California, Irvine (UCI) Genomic High-Throughput Facility. The libraries were constructed using the PacBio SMRTbell template prep kit 1.0, annealing of the sequencing primer was done according to PacBio guidelines, and sequencing was performed using the DNA/polymerase binding kit P5 and the PacBio DNA sequencing reagent 3.0. The high-throughput sequencing yielded 268,823 reads, totaling 855,362,482 bp.

The sequencing reads were assembled using the PacBio SMRT Analysis software version 2.3.0 into 2 high-quality contigs (chromosome I, 3,197,498 bp; chromosome II, 1,618,489 bp), with 40% G+C content, a mean coverage of 138×, and 99.99205% consensus accuracy. A preliminary annotation of the genome was conducted using the NCBI Prokaryotic Genome Annotation Pipeline and the Rapid Annotations using Subsystems Technology (RAST) server (11), which resulted in the identification of 4,390 genes, 94 tRNAs, and 22 rRNA coding sequences. To our knowledge, this is the first published complete Pseudoalteromonas genome sequence that was isolated from a diseased coral colony.

### Nucleotide sequence accession numbers.

This whole-genome shotgun project has been deposited at GenBank under the accession numbers CP009888 and CP009889, representing chromosomes I and II, respectively.
